# What Is the StartReact Effect?

**DOI:** 10.1111/apha.70235

**Published:** 2026-05-04

**Authors:** Anthony N. Carlsen, Dana Maslovat

**Affiliations:** ^1^ School of Human Kinetics University of Ottawa Ottawa Ontario Canada

**Keywords:** motor preparation, reaction time, reticulospinal, startle, StartReact

## Abstract

The use of a startling acoustic stimulus during a simple reaction time task results in the rapid initiation of a prepared response at extremely short latencies (< 80 ms). This so‐called “*StartReact effect*” has been increasingly employed to probe subcortical contributions to response preparation, as it is thought to occur due to increased activation in reticulospinal pathways associated with engagement of the startle reflex. However, the lack of an agreed‐upon definition of what exactly constitutes a StartReact effect, combined with differences in methodological protocols, has resulted in inconsistent interpretation of experimental results. Based on a comprehensive review of the literature, including evidence for the physiological mechanism underlying the effect, we propose that the clearest definition of the StartReact effect is “the early and involuntary triggering of a prepared movement in the presence of a startle reflex”. Reflexive startle activity has been shown to be strongly associated with involuntary response initiation and avoids other potential confounding variables that have been shown to speed reaction time. Here we argue that classification of trials based on startle‐related activation in sternocleidomastoid is the most robust method to confirm a StartReact effect. Special situations, such as pre‐pulse inhibition, movements involving musculature that require additional considerations, and lowered response preparation levels, are also considered with regards to how to confirm the presence of a StartReact effect. Future directions, including the use of a StartReact protocol as a potential adjuvant therapy for movement disorders, are discussed.

## 1 Origins and Importance of the StartReact Effect

In a series of studies by Valls‐Solé and colleagues [1, 2, 3, 4], participants performed several simple reaction time (RT) tasks whereby a known response (e.g., wrist extension, rise to tiptoes) was executed as fast as possible following the presentation of a visual go‐signal. Critically, on several random trials the go‐signal was accompanied by an extremely loud (~130 dB) auditory stimulus that was generated by discharging a magnetic stimulation coil over a metal plate. This unexpected intense auditory stimulus resulted in a reflexive startle response, but also led to the planned reaction time task responses being produced at extremely short latencies (< 80 ms) that were much quicker than those made in response to the typical go‐signal. The observed reduction in reaction time was far larger than would be expected by the well‐researched effects of increased stimulus intensity [5] or intersensory facilitation [6], and in some participants, the response latency was shorter than the calculated minimum time for cortically‐mediated response transmission [3]. This was a surprising result, and because the pattern of muscle activity seen in the electromyography (EMG) of these extremely short‐latency responses was unchanged on startle trials, the authors concluded that voluntary reactions were not just appended on to the earlier startle reflex, but that the prepared responses appeared to be “triggered entirely by activity at subcortical levels, probably involving the startle circuit” [3, p. 931]. Anecdotally, participants also reported that on trials where a startling acoustic stimulus (SAS) was presented, the prepared response seemed to be initiated by something other than their own will, which implied that it was the startle itself that seemed to trigger the prepared reaction. This phenomenon has become known as the “StartReact” effect. Indeed, the first known published use of the term was in 1998 where the authors describe the StartReact effect as “the startle‐induced reaction‐time‐shortening effect that occurs when an SAS is applied together with the ‘go’ signal” [2, p. 1315].

The discovery of the StartReact effect has intrigued researchers from a variety of disciplines, as it appears to involve a complex relationship between reflexive and voluntary neurophysiological motor pathways, which is not fully understood. Consequently, there has been a steady increase over the past quarter‐century in the use of a StartReact protocol to examine the control of movement. Although originally used as a means to probe which aspects of a response could be pre‐programmed (see [7] for a review), it has also been used to examine how movement preparation changes with practice [8], and more recently to investigate the relative contributions of corticospinal and reticulospinal pathways to movement preparation and initiation [9, 10, 11]. As mounting evidence suggests that the execution of movement involves a network of neural pathways at the cortical, brainstem, and spinal levels, researchers have considered whether it is possible to promote the use of undamaged/preserved circuitry that is engaged by the StartReact effect in movement‐disordered populations to recover a greater degree of motor function. For example, the rapid facilitation of a prepared response through the use of a StartReact protocol has been proposed as a potential rehabilitation protocol for Parkinson's Disease [12, 13], stroke recovery [14, 15, 16], and even less common neurological conditions such as Brown‐Séquard Syndrome [17], owing to its potential ability to bypass normal voluntary initiation circuits to trigger motor responses via subcortical reticular pathways. Reticulospinal pathways have been recently implicated as having an important role for strength gain and motor recovery (see [18, 19] for reviews), making the StartReact protocol an important potential adjuvant therapy for movement disorders. However, as the use of the StartReact protocol becomes more commonplace and applied in a greater variety of situations, it is critical to ensure there is an accepted definition of what comprises the StartReact effect and how the presence of the effect is confirmed, as this can influence the interpretation of any experimental results.

## 2 What Exactly Is the StartReact Effect?

Although the presentation of a loud acoustic stimulus is increasingly being used as a tool to examine response preparation, initiation, and execution across a variety of populations, there is not an accepted definition of what constitutes the StartReact effect. For example, some authors have assumed a StartReact response to have occurred whenever an intense stimulus was presented in a reaction time task [20], while others have utilized various secondary indicators such as the presence of a startle reflex [21, 22] or the magnitude of reaction time shortening [23]. Indeed, experimental methods for recent studies employing a StartReact protocol have differed quite dramatically in terms of the acoustic parameters of the loud stimulus and inclusion/exclusion criteria for what is considered a “good” StartReact trial (see [24] for a review and recommendations). These considerations and classifications become even more critical when the StartReact protocol is being evaluated as a rehabilitation tool to improve volitionally activated reaching movements in movement disordered populations (e.g., [16]).

Defining the StartReact phenomenon has been challenging, and oftentimes controversial, primarily because of the disagreement about the neural pathways hypothesized to underlie the effect (see Section 3 below). Nevertheless, one common element, irrespective of these pathway considerations, is that the intense stimulus consistently acts to somehow trigger the planned voluntary response early and *involuntarily*, provided there is sufficient preparatory activation prior to the intense stimulus being presented (see [7] for a review). Indeed, several studies have shown that the presentation of an irrelevant, intense acoustic stimulus can trigger a precued response prior to the typical visual go‐signal in a reaction time task, and this early triggering occurs with increasing probability as the auditory stimulus approaches the go‐signal [25, 26, 27, 28]. This involuntary response initiation is an important component of defining the StartReact effect, as it distinguishes this phenomenon from the more gradual and linear reaction time reductions caused by intersensory facilitation [6] or stimulus intensity effects [5]—both of which act to speed up cortically‐mediated voluntary initiation. Many studies (including a meta analysis; [29]) have concluded that trials with startle reflex related activity are triggered early more often and/or produced at latencies significantly shorter than those same intense stimulus trials without startle reflex activity (e.g., [27]). Thus, we propose that a definition of the StartReact effect that best encapsulates the experimentally described phenomenon, yet is not dependent upon a specific neural mechanism, is “early and involuntary triggering of a prepared movement in the presence of a startle reflex.”

### 2.1 Confirmation of Startle and the StartReact Effect

Some potential difficulties arise from the proposed definition for the StartReact effect, since it may be challenging to verify whether a response has been involuntarily “triggered” or voluntarily initiated, and in many cases, this must be determined as secondary to a related phenomenon. Originally, the StartReact effect was confirmed based on the presence of startle reflex activity, as the triggering of responses was considered to be tied to engagement of startle circuits [3]. Many other studies have adopted this approach, whereby only trials showing startle reflex‐related activation are considered to be involuntarily triggered and thus included in the StartReact analysis [21, 22, 30]. In order to establish that a startle reflex has been elicited, various muscular markers have been used, with the most common being a short latency burst of EMG activity in orbicularis oculi (OOc) and/or in the sternocleidomastoid (SCM) (see [24] for a review). Typically, SCM activation has been favored due to its higher sensitivity as a startle indicator. More specifically, the probability of eliciting a burst of EMG in SCM has been shown to be strongly affected by stimulus intensity, with less than 20% of trials showing this startle reflex activation when the intensity is 100–104 dB and steadily increasing to above 80% in healthy young adults when the intensity is ≥ 120 dB [21, 27, 31]. On the other hand, OOc activity does not show similar fidelity in differentiating between intensities, with a large majority of trials showing OOc activity at intensities as low as 100 dB [32, 33]. Furthermore, it has been suggested that OOc activity may occur as an auditory blink reflex in the absence of a more complete startle reflex [21, 32].

One potential drawback to using the presence of SCM activity to confirm a startle reflex is related to its reliability at a population level. It has been reported that SCM activation following an intense stimulus may not be elicited (or detectable) in 20% or more of healthy participants [24, 32, 34], and the rate of non‐responders may be higher or more variable in clinical populations such as individuals with Parkinsonian syndromes [35, 36, 37] or chronic stroke [38, 39]. The lack of an observed burst of EMG in SCM following an intense acoustic stimulus may be due to difficulty in detecting the response in the recorded EMG signal, or to differential response thresholds in some individuals [40]. Thus, it has been argued that using SCM as an indication that a startle reflex has occurred may underestimate the true incidence of the startle reflex [41].

An alternative approach has been to classify *all* reactions following a loud acoustic stimulus as StartReact responses [20, 42, 43, 44]. On one hand, this assumes that the response is always at a sufficiently high state of preparation for triggering to occur, which may not be the case owing to experimental design or an inattentive participant. More potentially problematic is that this approach assumes that the StartReact effect is “stimulus‐driven,” and that *any* intense stimulus will always involuntarily trigger a prepared response. However, this adds another confounding variable to the determination as to whether a response has been triggered, as it is unclear whether there is a *minimum threshold* for a stimulus to involuntarily trigger a prepared reaction. This is especially relevant as some studies that have claimed to be investigating the StartReact effect have employed stimulus intensities as low as 100–110 dB [45, 46, 47], which typically only elicit a startle reflex on a relatively low proportion of trials (e.g., 15%–40%) [31]. In short, assuming that all loud stimulus trials will elicit a “StartReact” response does not allow for a distinction between involuntary response triggering per se and reductions in reaction time latency due to stimulus‐driven effects on the voluntary response, such as stimulus intensity and intersensory facilitation [5, 6].

In order to correctly categorize responses as triggered or non‐triggered, another suggestion has been to calculate a cumulative distribution function (CDF) of the reaction time latency for acoustic stimulus trials [23]. The rationale for this approach is that trials with shorter reaction time latency are more likely to have been triggered. As such, the data are sorted by reaction time quantiles and the fastest onset trials (0%–45% quantiles) are considered to be involuntarily triggered, whereas the slower onset trials (55%–100% quantiles) are considered to be voluntarily initiated. The authors suggested that a CDF analysis allows for inclusion of trials regardless of whether SCM activation occurs, resulting in a lower burden to the participants in terms of needing to use a more intense stimulus or to collect additional trials. However, similar to the pitfalls introduced by including *all* loud stimulus trials, this method does not account for stimulus intensity differences, as it would be expected that the number of involuntarily triggered responses following a 100 dB versus a 130 dB stimulus would be substantially different, and thus including the top 45% of fastest reaction time trials would not correctly categorize StartReact trials in either condition.

**FIGURE 1:**
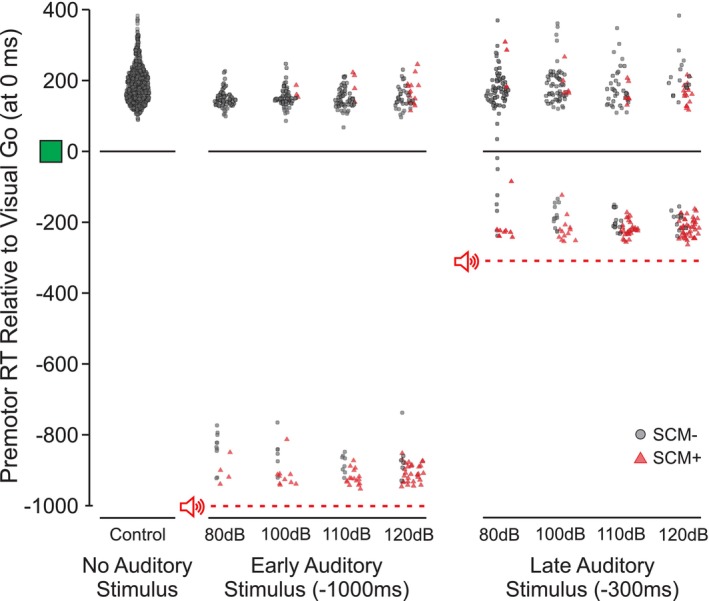
Individual trial premotor reaction time (RT) as a function of stimulus condition, time locked to visual go‐signal onset (data from [27]; originally published as means and histograms). Acoustic stimuli of varying intensities were presented either 1000 ms (early) or 300 ms (late) prior to the visual “go” (shown with green square), which occurred at 0 ms (acoustic stimulus presentation times shown with red horizontal dashed lines). Individual trial reaction time data are shown for the control condition (no auditory stimulus) or when an auditory stimulus was presented, which were categorized depending on whether a startle reflex response in sternocleidomastoid (SCM) was observed (red triangles, SCM^+^) or not observed (gray circles, SCM^−^). Note that for the auditory stimulus trials, a large majority of reaction times that occurred prior to the visual go‐signal were accompanied by SCM activity, and that the proportion of these trials increased with increasing acoustic stimulus intensity.

**FIGURE 2:**
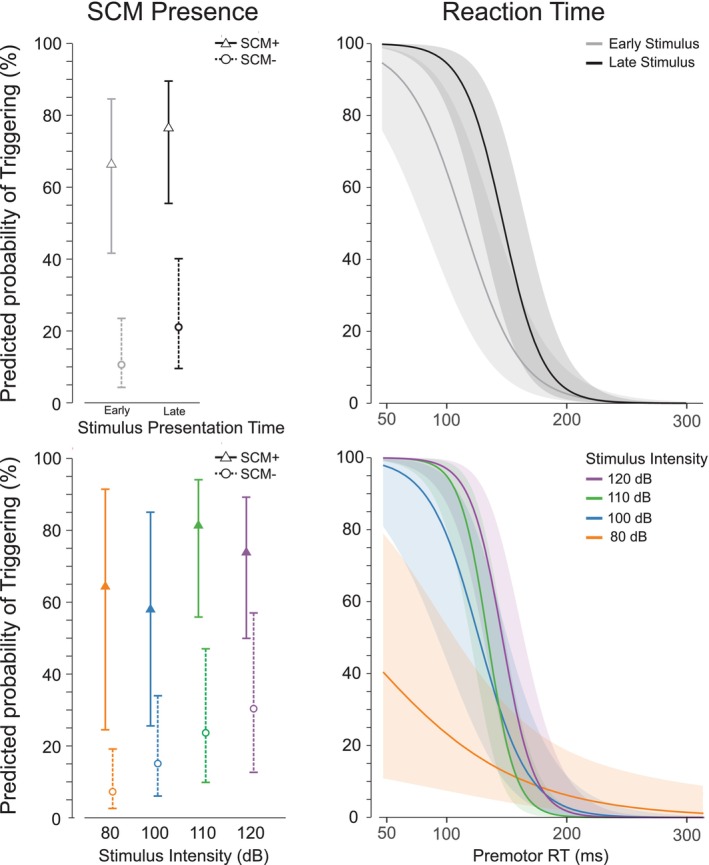
Predicted probability of observing response triggering as a function of different classifiers and covariates (adapted from [27]). Left panels show probability of response triggering as a function of the presence (triangle markers) or absence (circle markers) of startle‐reflex related activity in sternocleidomastoid (SCM). Right panels show probability of triggering as a function of premotor reaction time (RT). The top panels show probability with the stimulus presentation time as a covariate (early = the stimulus was presented 1000 ms prior to the go‐signal; late = 300 ms prior to the go‐signal), whereas the bottom panels show probability expressed with stimulus intensity as a covariate. Note that the predictive power of SCM presence is relatively high and largely unaffected by presentation time or stimulus intensity, whereas the predictive power of reaction time substantially declines at lower stimulus intensities and earlier stimulus presentation times.

To evaluate the different StartReact trial inclusion strategies, a study was specifically designed to examine under what conditions response triggering occurs [27]. In this study, participants performed a simple reaction time task, in which an irrelevant auditory stimulus of varying intensity (80–120 dB) was presented when participants were either in a low preparatory state (1000 ms before the visual‐go signal), or a high preparatory state (300 ms before the go‐signal). By presenting the auditory stimulus in advance of the go‐signal, responses were considered to be triggered involuntarily if they were initiated before the visual imperative signal occurred (but after the auditory stimulus), whereas responses were considered to be voluntarily produced if they were initiated following the visual go‐signal. As expected, the incidence of response triggering by the auditory stimulus increased with both stimulus intensity and preparation level (i.e., later stimulus presentation time). However, the early responses were predominantly associated with the presence of startle reflex activity in SCM (Figure 1). Importantly, logistic regression also confirmed that the strongest discriminator of involuntary triggering by the acoustic stimulus was the presence of a startle reflex identified via short latency activation in the SCM (as compared to reaction time or stimulus intensity alone). Furthermore, the extent to which the presence of SCM activation was associated with response triggering was not influenced by stimulus intensity or presentation time, whereas the association between reaction time and response triggering was significantly diminished for lower stimulus intensities and early presentation times (Figure 2).

### 2.2 Identifying a StartReact Effect Using SCM Activation

The data from Maslovat et al. [27] strongly support the proposal that the most valid and reliable approach to identify StartReact responses is to classify trials based on the presence/absence of startle reflex activity in the SCM (i.e., SCM^+^ or SCM^−^), and to consider those trials exhibiting a startle reflex (SCM^+^) to be involuntarily triggered. First, the use of this method is consistent with previous data showing that there is a robust and significant decrease in response onset latency of the intended action in SCM^+^ versus SCM^−^ trials, even when stimulus intensity is controlled [9, 23, 29]. Second, this method demonstrated that SCM activation can be used to correctly classify StartReact trials, even when preparation levels were substantially different. Although lowered preparation level typically results in fewer SCM^+^ trials, it has been shown that the reported SCM^+^/SCM^−^ reaction time differences are not simply caused by differences in preparation level, but instead involve qualitatively different processes [48]. Finally, the results observed using this method support previous studies that have argued that the additional reaction time shortening observed on SCM^+^ trials is associated with overt neural activation in reticular formation, over and above any reaction time facilitation (relative to non‐startle trials) seen in SCM^−^ trials due to stimulus intensity or intersensory facilitation effects.

The inclusion of only SCM^+^ trials when quantifying the StartReact effect also addresses the confounding issue of stimulus intensity, as the probability of a stimulus eliciting a startle reflex is strongly related to the intensity of the stimulus. Without confirmation of an overt startle reflex, there is no accepted lower limit to what intensity of stimulus can be considered appropriate for use in a StartReact protocol. For example, the use of a 90 dB tone would be very unlikely to elicit a startle reflex in SCM, yet would show a reduction in reaction time relative to an 80 dB acoustic go‐signal [21]. This illustrates a potential pitfall of using a CDF approach, where it is assumed that the fastest 45% of reaction times are indicative of trials in which response triggering occurred [23], as this approach would have the effect of erroneously *including* many trials not exhibiting a StartReact effect when a lower intensity “startling” stimulus was used. Furthermore, this would also *exclude* many trials actually exhibiting the StartReact effect at high stimulus intensities, as response triggering has been shown to occur on > 80% of trials using a 120 dB stimulus [27]. Alternatively, simply including all “loud stimulus” trials versus inclusion/exclusion of trials based on the presence/absence of SCM activity can lead to very different conclusions about response preparation and initiation processes. Indeed, one study that employed a 114 dB acoustic stimulus but did not exclude loud stimulus trials that lacked an SCM response concluded that activation related to response preparation increased just prior to the go‐signal [44]. However, when this study was replicated and the data were reanalyzed with the StartReact condition including only SCM^+^ trials (which only occurred on 28% of the total loud stimulus trials), it was found that movement‐related activation was instead constant prior to response initiation [49].

**FIGURE 3:**
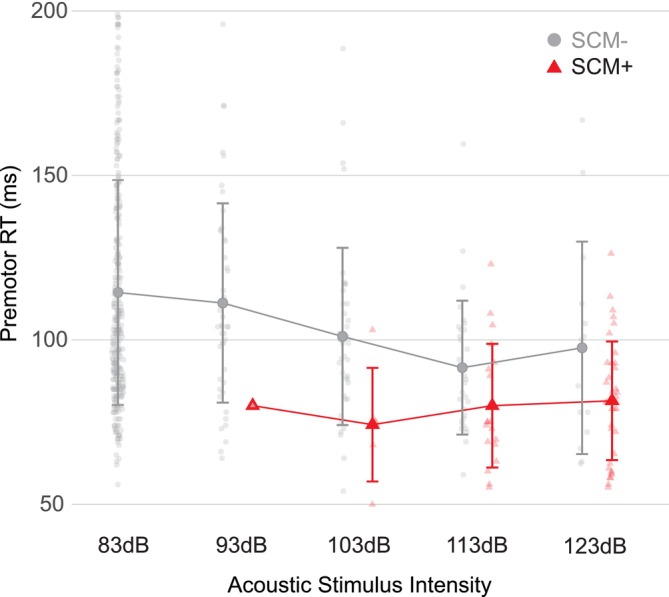
Premotor reaction time (RT) as a function of stimulus intensity, separated based on whether a startle reflex in sternocleidomastoid (SCM) was observed (SCM^+^, red triangles) or was not observed (SCM^−^, gray circles). Originally published as participant mean data [21]; here, individual trial data points (small markers) are plotted along with grand means (large markers) and SD (whiskers). Data are truncated at a maximum reaction time of 200 ms for visibility (loss of 13 data points from the 83 dB condition). Note that SCM^+^ trials show a similar reaction time for all intensities, which is significantly lower than SCM^−^ trials, which show a linear decrease in reaction time as intensity increases.

Thus, the preponderance of the evidence suggests that for studies employing the StartReact effect, the confounds of stimulus intensity and/or different states of motor preparation can be avoided when SCM activation is used as an inclusion criterion for identifying StartReact responses. SCM^+^ trials not only robustly predict response triggering, but also typically exhibit similarly short absolute response latencies, irrespective of stimulus intensity and/or preparation level. This relationship does not occur for SCM^−^ trials, which show a much stronger linear relationship between increasing intensity and decreasing response latency, yet never attain a similar degree of reaction time reduction as that seen in SCM^+^ trials, even at the highest intensity levels (Figure 3). As noted above in Section 2.1, one downside of using startle reflex‐related activation as an inclusion criterion is that there may be fewer trials that elicit a SCM^+^ response when lower intensities are employed [21, 27, 31]. This may necessitate the exclusion of *all* trials from the relatively small proportion of participants who do not show measurable SCM activation, as it is currently unknown whether these individuals are also immune to the StartReact effect. Nevertheless, using SCM^+^ trials as confirmation that a StartReact effect has occurred allows for flexibility in methodological approaches, especially in the intensity of the auditory stimulus used, while still preserving the integrity of the effect of interest.

It should be acknowledged that the association between startle‐related SCM activation (SCM^+^) and involuntary response triggering is not an all‐or‐none relationship, as some voluntarily‐initiated trials might include SCM activation, and some trials that are involuntarily triggered may lack SCM activity (see Figure 1). Data from the study by Maslovat et al. [27] investigating response triggering by a loud stimulus allowed for quantification of false positives (i.e., SCM^+^ trials incorrectly identified as triggered) and false negatives (i.e., SCM^−^ trials incorrectly identified as voluntarily initiated). The incidence of these classification errors was relatively low for all stimulus intensities and preparation levels (< 20% total for most conditions) [27], figures 9 and 12, and thus would appear to have only a negligible effect on biasing the data. This is in direct contrast to the previously described alternative classification approaches, which are likely to have a substantially greater number of erroneously classified trials, which would be magnified as the stimulus intensity and/or preparation level decreases. It should also be noted that the observed relationship between SCM activation and the reaction time facilitation that characterizes the StartReact effect does not imply that the SCM activity *causes* the StartReact effect. Rather, it is proposed that underlying activation, likely in reticular formation, is responsible for the expression of both responses.

**FIGURE 4:**
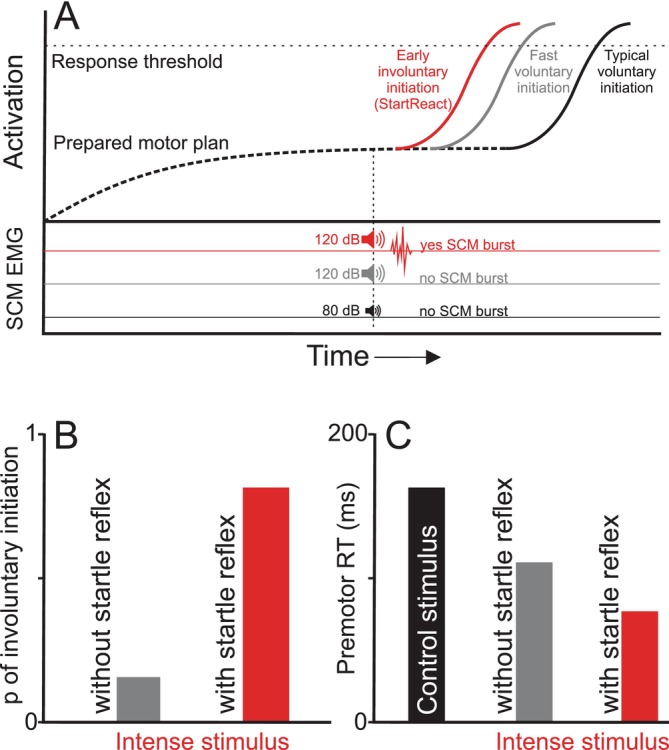
Schematic representation of the StartReact effect. Panel A depicts neural activation level (black dashed line) as a function of time in a simple reaction time paradigm, where activity rises above baseline as a voluntary motor response is prepared. Following the acoustic “go” signal (80 or 120 dB), activation then rises to cross the response initiation threshold (gray horizontal dotted line). Startle reflex electromyographic (EMG) activity in sternocleidomastoid (SCM) is shown at the bottom of panel A. Typical voluntary initiation following an 80 dB acoustic stimulus is shown in black (no EMG activity in SCM). Fast voluntary initiation following an intense 120 dB stimulus (due to the effect of stimulus intensity and/or intersensory facilitation) is shown in gray (with no activity in SCM). Early i*nvoluntary* initiation following a 120 dB stimulus, accompanied by EMG activity in SCM is shown in red (i.e., the StartReact effect). Panel B illustrates the probability of observing involuntary response triggering following an intense (120 dB) stimulus as a function of the observed presence (red) or absence (gray) of startle reflex‐related EMG activity in SCM (data adapted from [27]). Panel C depicts typical premotor reaction time (RT) observed on typical voluntary (control) trials (black), as well as on trials following an intense acoustic stimulus where a startle reflex is (red), or is not observed (gray).

In summary, a comparison of the various classification approaches for trial inclusion in StartReact studies suggests that SCM activation is the most robust and accurate predictor of involuntary response triggering, regardless of stimulus intensity or participant preparation level. In addition, this approach is congruent with the explanation that engagement of the startle reflex circuitry results in a quantitatively different response that is initiated through a faster and involuntary mechanism. Figure 4 schematically illustrates this difference between responses that are not triggered by an intense stimulus, yet nonetheless exhibit shorter reaction times via cortical processes such as stimulus intensity facilitation and intersensory facilitation (gray), and those thought to be involuntarily triggered via activation in reticular structures that is confirmed by an overt startle reflex (red). Indeed, the “Start” in the nomenclature of “StartReact” was originally derived from observed activation in *startle* reflex‐related circuitry, which was thought to be a necessity for involuntary response triggering to occur [3]. Therefore, we argue that in the vast majority of situations, it is imperative to confirm the presence of a startle reflex to be assured of overt neural activity in startle‐related circuits that strongly predicts involuntary triggering by an intense stimulus (i.e., the StartReact effect). The assertion that the presence of a startle reflex is necessary to confirm the StartReact effect necessitates a discussion of the proposed physiology underlying the effect.

## 3 Proposed Physiology Underlying the StartReact Effect

The original explanation for the rapid release of a prepared response that characterizes the StartReact effect proposed that the prepared movement was somehow triggered through the involvement of subcortical brainstem circuitry common to startle reflex and voluntary response pathways [3]. The reticular formation was posited to be the locus of this interaction as it is known to contribute to voluntary motor function, but more critically it is the structure primarily responsible for the generation of the startle reflex. Since then, numerous studies have linked reticular activation (primarily indexed via an overt startle reflex) with the presence of a StartReact response, which is different from the simple speeding effect of stimulus intensity on reaction time. Therefore, it has been proposed that in StartReact experiments where the intended voluntary response is triggered at short latency following a startle stimulus, reticular activation generated from presentation of the loud acoustic stimulus also acts to trigger the planned action that is (at least partially) represented at near‐threshold levels in adjacent reticular structures [9]. This representation could consist of a subcortical analog of cortical cell assemblies proposed by Wickens et al. [50] as a mechanism to explain motor programs. As such, on these trials a large proportion of the descending drive that contributes to the production of the prepared and intended movement may originate from activation of reticular structures that contribute to voluntary movement production.

### 3.1 Reticular Contributions to Startle and Voluntary Responses

The mammalian startle reflex is a brainstem‐mediated, protective response that is characterized by a diffuse set of short latency muscular contractions, as well as changes in heart rate and skin conductance, and is typically elicited by an intense and unexpected stimulus [51]. In the case of an intense acoustic stimulus, the circuit involves direct and indirect projections from cochlear nucleus to the giant neurons of the caudal pontine reticular formation. These neurons conduct along the reticulospinal tract to various levels of the spinal cord and act as command neurons responsible for generation of the motor activation that characterizes the startle reflex (see [51]). Importantly, these neurons require strong synchronized or temporally summed input in order to fire due to their morphological and electrophysiological properties [52, 53]. This results in an increasing probability of eliciting a startle reflex with increasing acoustic stimulus intensity [21, 51]. Once a startle reflex is elicited, the motor components of the startle response are typically elicited in a rostral to caudal pattern with the earliest and highest probability response (aside from the acoustic blink reflex) being short latency activation seen in the sternocleidomastoid and masseter muscles [32].

In addition to the startle reflex, it has been demonstrated that the reticular formation also contributes to voluntary motor output. This has been shown through the use of direct neural recordings in higher order mammals such as cats [54], as well as in non‐human primates [55]. Because of the nature of the descending outputs, it has been generally accepted that the reticulospinal system in humans acts predominantly on axial and proximal limb musculature and contributes more prominently to the control of muscle tone, posture, and stereotyped action such as walking [56, 57]. However, it has been shown that the reticular formation receives extensive descending connections from the cerebral cortex, including direct corticoreticular fibers from primary motor cortex and collaterals of corticospinal neurons, as well as descending connections from premotor areas such as premotor cortex and supplementary motor area [58, 59, 60]. Thus, the reticular formation likely plays a more prominent role in voluntary movement control than originally thought (see [11]). The output of the reticular formation is strongly bilateral and reticulospinal tracts primarily contribute to the medial descending system, but because it interacts with many other brain regions and systems, it is difficult to understand its functions in isolation. This is further complicated by the reticular formation exhibiting inhibitory zones in the medial part of the medulla, and facilitatory areas in the lateral medulla and caudal pons (although during normal activity these effects fluctuate) [61]. More recently, however, converging evidence has implicated the reticular formation in the production of fine motor actions in more distal musculature [62]. For example, recent extracellular recordings from the reticular formation in macaque monkeys showed stronger than anticipated connections to distal musculature and greater than expected activity during distal upper limb control [11]. Indeed, a study examining a variety of upper and lower body muscles, including flexors and extensors in both proximal and distal limbs, found the presence of reticulospinal drive in all examined muscles, indicating a widespread role of these pathways in human motor control [17].

### 3.2 Potential Physiological Mechanisms of StartReact

Although the StartReact effect was originally proposed to arise as a result of the involuntary triggering of a planned voluntary response via reticulospinal pathways [3], the precise mechanism underlying this explanation has been a matter of much debate [7, 9, 10, 11, 41, 42, 63]. Early evidence supported the assertion by Valls‐Solé et al. [3] that the planned movement was represented in subcortical structures and “released” by the startle. While StartReact studies have predominantly shown the effect in speeding upper limb movements, numerous studies showed that reaction time for a variety of other movement types and effectors could also be substantially facilitated by startle, including step initiation [26, 64], eye movements [65], and head rotations [66]. Furthermore, it was demonstrated that distal effectors such as intrinsic hand muscles, which have been thought to be primarily mediated by corticospinal connections and to be lacking significant reticulospinal projections, showed negligible reaction time facilitation by the startle, while other more axial movements that receive larger amounts of reticulospinal input showed a much more substantial degree of facilitation by the startle [39, 42, 63, 67]. More evidence for this “subcortical storage and release” mechanism was provided by a study involving patients with an inherited motor disorder known as hereditary spastic paraplegia (HSP). These individuals typically exhibit weakness and delayed reaction times in the lower limb, resulting from degeneration of corticospinal tracts. However, in these patients, lower limb reaction time latencies following a startle stimulus were accelerated to such a degree that they were similar to those seen in non‐HSP (control) participants [68], a result attributed to the response being transmitted via intact reticulospinal pathways. Similarly, it has been shown that startle can facilitate both the initiation and production of reaching movements using the paretic arm in patients with moderate to severe stroke [16], suggesting a subcortical locus of the effect.

Although there was robust evidence for this subcortical (reticular) storage hypothesis, there was little physiological evidence supporting a specific mechanism by which this would occur. As such, several alternative mechanisms were suggested that involved the normal cortically‐mediated movement production circuitry. For example, it was alternatively hypothesized that the reaction time facilitation produced on startle trials was simply an extreme version of the well‐known stimulus intensity effect [69] and involved the same cortical pathways used in voluntarily initiated responses. In this explanation, the reaction time facilitation is caused by the loud stimulus acting as an accessory stimulus to drive response‐related cortical activation levels above the initiation threshold [41]. Another alternative proposed mechanism underlying the StartReact effect was that movements were cortically planned and represented, but that following a startle, ascending activation (via the reticular activating system) provided sufficient excitation to the initiation centres to lead to the early and involuntary release of the planned movements—albeit via the normal corticospinal descending pathways [7]. This “reticular triggering” hypothesis was initially proposed following the observation that when transcranial magnetic stimulation (TMS) was used to induce a cortical silent period (which delays the onset of voluntary reactions), it also led to the delay of startle‐triggered responses [25, 70]. This suggested that regardless of the trigger source, the prepared response was carried out via the same corticospinal circuitry. However, it became clear that these cortically‐oriented mechanisms could not completely account for some relevant findings. For example, this could not explain the reaction time facilitation in patients with HSP [68]. In addition, it was demonstrated that the duration of the reaction time delay induced by TMS on StartReact trials was significantly smaller than that on control trials, implicating that there must exist some additional alternative neural drive during startle‐triggered movements [71]. Overall, the preponderance of the evidence suggests that StartReact responses do not simply represent cortically prepared and stored motor plans that are triggered by ascending reticular activation but alternatively, appear to involve direct descending drive from subcortical areas, likely reticular formation.

Recent evidence has shown that a large proportion of the early descending drive on StartReact trials may indeed originate from non‐cortical centres. One recent study recorded high density EEG while participants performed wrist extension movements and showed that while movement related cortical potentials (time locked to EMG onset) were consistently observed on non‐startle trials, this same cortical activity was absent when the response was triggered at short latency by a startling stimulus [72]. Similarly, Tapia et al. [11] trained 2 macaque monkeys to perform arm flexion and extension movements in an instructed delay reaction time task while collecting extracellular recordings from primary motor cortex and reticular formation neurons. These authors found that both cortical neurons and reticular neurons fired selectively for flexion or extension movements on typical reaction time trials. On startle trials, reaction times were significantly speeded, while cortical activity was in fact diminished in the short interval following a startle evoking stimulus. On the other hand, reticular drive was upregulated in the 70–80 ms following the startle while becoming slightly less specific to the prepared response [11]. Based on these results, they asserted that the StartReact effect may arise from the startle providing *nonspecific* reticular drive to the motorneuron pool that is additive with the descending cortical drive, which maintains sufficient levels to ensure specificity of the voluntary response. While this assertion is congruent with previous research that suggested an additive effect of startle‐related drive with other voluntary descending drive [73, 74], these latter authors did not speculate on the specificity of the reticular drive.

**FIGURE 5:**
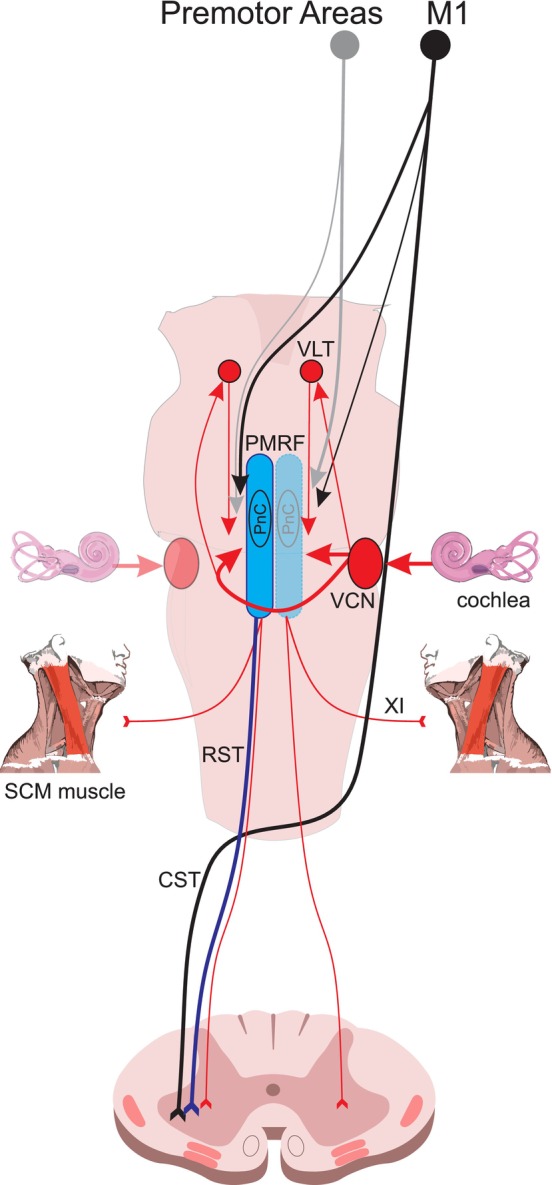
Schematic depiction of proposed pathways involved in the StartReact effect. Brainstem nuclei of the pontomedullary reticular formation (PMRF, shown in blue) receive bilateral inputs from primary motor cortex (M1; black lines) as well as from premotor areas such as premotor cortex and supplementary motor area (gray lines). M1 connections to the contralateral PMRF are generally stronger while ipsilateral connections from premotor areas are stronger (denoted by line thickness). The startle reflex pathways are shown as red lines (unilaterally for clarity). Following a sufficiently intense auditory stimulus, input from cochlea activates the ventral cochlear nucleus (VCN), which projects bilaterally to PMRF (primarily the caudal pontine reticular nucleus, PnC) via direct and indirect connections (e.g., through the ventrolateral tegmental nucleus, VLT). Activation of the PnC leads to descending startle reflex activity via the reticulospinal tract (RST, red lines) and activates the sternocleidomastoid (SCM) muscle via cranial nerve XI. For StartReact responses, it is proposed that voluntary activation descends via the corticospinal tract (CST) and converges at the spinal level with involuntary task‐specific descending drive (blue line) from the PMRF along the RST, together with some additional non‐specific activation associated with the startle reflex. These converging inputs are integrated at the spinal motor neuron pool, resulting in the early release of the prepared movement characteristic of the StartReact effect.

While the preceding data and their interpretation are compelling, it is nevertheless possible that the reticular drive elicited in the presence of a startle—that is likely additive with a diminished cortical drive at the level of the motorneuron pool—is in fact still highly specific to the prepared task. That is, while reticular activation that results in the startle reflex clearly elicits non‐specific bilateral drive, there may also be additional reticular drive that is specific to the voluntary action when a response is highly prepared (see Figure 5 for a schematic depiction of proposed pathways). As noted above, the reticular formation receives strong bilateral input from premotor areas such as SMA and premotor cortex [58]—areas that are known to be responsible for preparation of upcoming movements [75]. Indeed, some previous results argue in favor of this specificity interpretation. For example, it was demonstrated that when participants were trained to *relax* an ongoing contraction as fast as possible in a reaction time task, startle led to a much faster *offset* response in the EMG activity [76]. This suggests that the descending reticular drive did not simply activate the entire motorneuron pool to a greater extent, but that the drive acted predominantly on prepared inhibitory connections. Further evidence that the startle can result in reticular drive that is tuned to the needs of the prepared action comes from studies that have employed complex movements in a StartReact paradigm that required coordinated simultaneous activation and inhibition drive to various muscles. It might be expected that if the startle simply increased nonspecific reticular drive, these actions would be compromised to some extent by unwanted and abnormal co‐contraction driven by the startle reflex itself. However, this does not appear to be the case, as the timing and magnitude of the EMG burst patterns, as well as the resultant kinematics is largely unchanged in these complex tasks. For example, it was shown that when participants were preparing for a quick step initiation, presenting a startling stimulus led to short latency step initiation, including speeding of the anticipatory postural adjustments necessary for maintain postural equilibrium ([26]; see also [77] for a review). Similarly, it was shown that kinematics and EMG patterning in complex upper‐limb reaching movements to different targets were largely unaffected when a startling stimulus triggered the responses [78, 79].

It is important to note that the evidence for subcortical involvement does not rule out the contribution of cortical structures to movement preparation, but rather suggests that response preparation and initiation processes involve a variety of cortical and subcortical structures [9]. As the evidence accumulates implicating the reticulospinal pathway in the StartReact effect, it has become more commonly used as a surrogate measure for reticulospinal involvement in a given movement (e.g., [80]). For example the relative magnitude of reaction time speeding by a startling stimulus across effectors has been used to infer the relative amount of reticular drive contributing to the movement, with shoulder movements demonstrating a greater reaction time reduction as compared to bilateral finger abduction [81]. Similarly, it was shown that when a finger abduction task was performed bilaterally, it was more susceptible to reaction time facilitation than if the same movement was performed unilaterally [67].

In summary, the current evidence points to the likelihood that the StartReact effect arises from a convergence of cortical and task‐specific reticular drive at the motorneuron pool that results in early and “involuntary” initiation of a prepared voluntary response. This does not discount the fact that there is also likely to be nonspecific startle‐related drive contributing to the activation at the level of the spinal cord. As such, in light of this mechanism, and the fact that increased stimulus intensity non‐startling stimuli can result in decreased reaction time latencies, it seems prudent that in order to infer additional reticulospinal drive on StartReact trials, a measure of this drive (other than a speeding up of reaction time) should be confirmed. Here we suggest that this confirmation should be from an observed startle reflex in an independent indicator such as SCM.

## 4 Special Situations Involving the StartReact Effect

We have argued that for reaction time tasks that employ an intense stimulus to trigger a prepared movement, startle‐related SCM activation appears to be the most reliable indicator of the StartReact effect; however, there are some experimental situations that can pose challenges to measurement and confirmation of the StartReact effect which are outlined below.

### 4.1 Tasks Involving Prepulse Inhibition of Startle Reflex Activity

One experimental situation that can complicate a StartReact protocol involves the phenomenon of “prepulse inhibition,” whereby the presentation of a low‐intensity non‐startling stimulus just prior to the SAS can cause attenuation of the startle reflex [82]. It is important to note that the prepulse stimulus acts to attenuate rather than completely eliminate the startle reflex; yet, this can reduce the startle‐related burst of activity in the SCM to below the typical detection threshold, even though it may still be present and response triggering occurred. Prepulse inhibition is a specific situation in which the reticular structures may still be (partly) engaged and may also provide increased activation to the initiation circuitry, yet sensory gating acts to reduce the measurable output in the SCM muscle [83, 84]. Thus, when the presence of a prepulse stimulus is a necessary or unavoidable component of a StartReact protocol, the requirement of observing SCM activation as an inclusion criterion may result in a larger proportion of false negatives than would otherwise be observed (i.e., incorrect exclusion of trials where response triggering occurred). Research investigating prepulse inhibition during a StartReact protocol has suggested that the StartReact effect appears to remain mostly intact [85, 86]; however, these studies did not directly compare startle reaction times on prepulse trials with and without SCM activation, and thus it remains unclear if SCM^+^/SCM^−^ differences in reaction time are preserved or abolished due to these false negatives. Without this critical comparison, the most conservative and preferred method to ensure proper trial categorization in reaction time paradigms with this limitation is to use a sufficiently intense SAS (e.g., ≥ 120 dB) to increase the likelihood of eliciting a startle reflex even in the presence of prepulse inhibition, and then to only retain SCM^+^ trials. While it is currently unclear whether prepulse inhibition may sometimes result in a StartReact effect with attenuated or undetectable SCM activation, it still *cannot* be taken as evidence that confirmation of SCM activation is unnecessary in order to confirm the presence of a StartReact effect in *all* circumstances. Additional data regarding the effects of a prepulse on any potential reaction time differences between trials with and without SCM activity is needed prior to providing any alternative guidance.

### 4.2 Special Situations Involving Specific Musculature

In the StartReact literature, most studies have involved upper limb responses, which have consistently shown SCM^+/−^ differences in response latencies [29]; however, there are specific movement types that warrant special consideration. One such situation involves the previously discussed intrinsic hand muscles, which are thought to be primarily mediated by corticospinal connections. When unimanual finger movements are used in a StartReact protocol, earlier triggering of the prepared response on SCM^+^ trials (in comparison to SCM^−^ trials) is typically not observed. One explanation for this result is that there is less reticular activity that reaches distal upper limb musculature, likely due to decreased reticulospinal projections [32]. Thus, even though a startle reflex that evokes SCM activity may be observed when a SAS is presented during tasks involving more cortically‐mediated muscles, the amount of increased reticular activation provided to the pathways innervating these muscles may be limited, decreasing the likelihood of response triggering. Therefore, inclusion of these SCM^+^ trials as representative of StartReact responses when using unimanual finger responses would presumably constitute a large proportion of false positives. Indeed, when comparisons between strongly corticospinal and strongly reticulospinal movements are made, only the reticulospinal‐mediated responses show a reaction time *difference* on SCM^+^ trials. Put another way, while there is no further decrease in reaction time when SCM activity is present for corticospinal‐mediated movements, there is a substantial further reduction in reaction time when SCM activity is present in reticular‐driven movements. This effect has also been described when more cortically‐driven movements are combined with strongly reticular actions. For example, although SCM^+/−^ reaction time differences are not seen for unimanual finger‐lift movements, when these same movements are done in a bilaterally synchronous task, reaction time latencies are significantly shorter on SCM^+^ trials than SCM^−^ trials [67]. The explanation offered for this effect was that bimanual responses have been shown to involve greater reticulospinal drive than unimanual responses [87, 88], and thus were more susceptible to the StartReact effect. Similar results were demonstrated whereby combining a cortically‐driven finger‐pinch response with a more proximal elbow flexion movement led to SCM^+/−^ reaction time differences that were not otherwise present for the finger pinch alone [22, 89]. This result was attributed to additional reticular drive from the proximal musculature, which has been shown to involve more reticulospinal connections. Collectively, these results indicate that when strongly corticospinal‐mediated musculature is involved in a StartReact protocol, simply the confirmation of the presence of startle‐related SCM activation is *not* sufficient to confirm response triggering. Instead, a comparison of the reaction time latencies between SCM^+^ and SCM^−^ trials is necessary, with a significant reduction on SCM^+^ trials (as compared to SCM^−^) required to confirm the presence of a StartReact effect.

Another situation involves movements that directly or indirectly result in activation of the neck muscles including SCM, as these pose a challenge to disentangling startle‐related and voluntary response‐related SCM activity. These tasks may include postural perturbations resulting in rapid head displacement and associated corrections [90], large rapid movements of limbs requiring head stabilization [91, 92], or even direct head positioning tasks [66]. In these situations, careful analysis of SCM onset time can be used to distinguish between the responses, unless the onset windows overlap substantially. For example, in fast limb movements involving large musculature, anticipatory postural bracing in SCM is expected to be time‐locked to the onset of the limb movement, whereas startle‐related SCM activation is expected to occur within a defined time window following the intense stimulus (typically between 30 and 120 ms following the stimulus; [24]). In cases where these time windows overlap, it may be necessary to consider alternative (although potentially less reliable) startle reflex indicators such as masseter or orbicularis oculi [32], in addition to SCM.

Lastly, when considering studies involving lower‐limb effectors (e.g., stepping tasks), some have reported no difference in reaction times between trials with and without detectible SCM activity [38, 77, 93], while others have not investigated any potential association between the presence/absence of SCM activity and additional reaction time facilitation [13, 26, 68]. For the studies that have reported *no difference* in reaction times between SCM^+^ and SCM^−^ trials, it is possible that the reaction time effect in the lower limbs is simply smaller and thus these studies were simply not adequately powered to detect any potential difference. If this is the case, it does beg the question as to why the effect might be smaller in the lower limbs, and whether this may depend on the functional nature of the reaction time task. Specifically, it may be that although reticular formation is thought to contribute to the regulation of posture and gait [57], it may not be strongly involved in the preparation and execution of voluntary initiation in the lower limb. These complex interactions may be much more strongly mediated by cortical structures, particularly when postural requirements are high such as during step initiation [94]. However, given the relatively small number of studies using lower limb musculature, rather than assuming a lack of significant differences means that differences do not exist, it is clear that additional careful study is required to determine how task conditions might affect lower limb reaction time differences between trials with and without activation in SCM. Regardless, we suggest that in order to implicate reticular structures in the execution of a particular response, a conservative approach is to only include those trials where SCM activation was observed as these trials have an overt indication that there was increased reticular activation.

### 4.3 Situations Involving Lowered Response Preparation

A final special situation involves the use of StartReact protocols in paradigms or tasks where participants may be unable to achieve high levels of advance response preparation. For example, choice reaction time and dual‐task paradigms typically exhibit substantially longer reaction time latencies as compared to simple reaction time, which are attributed to a lower preparatory state [95, 96, 97]. When these protocols are implemented in a StartReact paradigm, SAS trials also often exhibit longer reaction time latencies as well as a lower incidence of SCM activation [98, 99]. The startling stimulus is thought to involuntarily trigger the planned movement by providing additional activation to overcome the initiation threshold, but this additional activation may not be sufficient to trigger the response when preparation levels are low and far from threshold [7]. Similar to the rationale provided for corticospinal‐mediated movements, in situations in which lowered preparation is expected, it is necessary to compare response latency between SCM^+^ and SCM^−^ trials to determine if the presence of startle‐related activation has an additional effect of further reducing response latency [99]. While the lack of any reaction time difference between SCM^+^ and SCM^−^ trials implies that a StartReact effect has likely not occurred, it is unclear if the presence of an SCM^+/−^ reaction time difference in this situation necessarily confirms that a StartReact effect has occurred. Given that all response latencies are considerably longer in these situations with lowered advance response preparation, the absolute response latency on SCM^+^ trials may not be sufficient to indicate that the response was involuntarily triggered by the SAS and could instead represent a speeded voluntary initiation process. At the present time, further study is warranted to clarify the interaction between the startle reflex and response initiation under these conditions of lowered response preparation level.

## 5 Conclusions and Future Directions

Over the past 25 years, the StartReact effect has become a valuable tool that can be used to evaluate processes and the neurophysiology associated with response preparation and initiation. Future studies will likely continue to clarify our understanding of the specific circuitry involved in the involuntary response triggering of a prepared response and allow for better understanding of the interaction between the startle reflex pathways and those involved in voluntary response initiation. While the weight of evidence currently supports a strong role of descending reticular drive in the production of StartReact responses, further confirmation of this involvement is warranted. If confirmed, the involvement of reticulospinal pathways in the StartReact effect would open up exciting possibilities in the treatment of movement disorders in which corticospinal pathways are degraded or damaged, and where the engagement of reticulospinal pathways may provide additional and/or alternative avenues for rehabilitation.

To facilitate the progression of this research, it is necessary to have a consistent definition of what constitutes a StartReact response and how this effect is confirmed. We argue that the clearest definition of the StartReact effect is: “The early and involuntary triggering of a prepared movement in the presence of a startle reflex”. Based on the literature reviewed, the most reliable indication of response triggering is through the presence of startle‐related SCM activation. This definition and inclusion criterion avoids the pitfalls of conflating voluntary reaction time facilitation with response triggering and allows for experiments that differ in methodological protocols, such as varying stimulus intensity and participant preparation levels. Although requiring the presence of SCM activation on startle trials may appear to be an overly conservative and somewhat cumbersome criterion, the benefit is that it provides confirmation of activation in reticular formation that is independent of response latency considerations. While it should be acknowledged that this may pose additional challenges for data collection involving clinical populations, given fewer eligible participants and limitations in the number of trial repetitions, confirmation of response triggering through involvement of reticular structures is even more critical in the examination of movement disorders, as it may form the basis of training and/or treatment interventions intended to target reticulospinal pathways. In order to minimize the rejection of trials without detectable SCM activity, the highest possible intensity auditory stimulus should be used (e.g., ≥ 120 dB) while taking care to minimize discomfort and injury risk [24]. Ultimately, a universally agreed‐upon definition of the StartReact effect should accelerate the goal of delineating the circuitry involved in this phenomenon and its potential application to understanding and improving human movement in both healthy and clinical populations.

## Author Contributions

**Anthony N. Carlsen:** conceptualization, writing – original draft, writing – review and editing, visualization, funding acquisition. **Dana Maslovat:** conceptualization, writing – review and editing, writing – original draft.

## Funding

This research was supported by grants from the Natural Sciences and Engineering Research Council of Canada (NSERC; RGPIN 2023‐04138) awarded to A.N.C.

## Conflicts of Interest

The authors declare no conflicts of interest.

## Data Availability

Data sharing not applicable to this article as no datasets were generated or analysed during the current study.
